# Acute abdomen in third trimester of pregnancy—a rare case of perforated Meckel’s diverticulitis

**DOI:** 10.1093/jscr/rjad492

**Published:** 2023-09-08

**Authors:** Jennifer Turco, Ihab Khalil, Mary Theophilus

**Affiliations:** Department of General Surgery, St John of God Public and Private Hospitals, Perth, WA 6000, Australia; Department of General Surgery, St John of God Public and Private Hospitals, Perth, WA 6000, Australia; Department of General Surgery, St John of God Public and Private Hospitals, Perth, WA 6000, Australia

**Keywords:** Meckel's diverticulitis, acute abdomen in pregnancy, appendicitis

## Abstract

Acute abdomen in pregnancy presents many diagnostic challenges. Non-specific symptoms, anatomical and physiological changes and diagnostic imaging limitations lead to diagnostic uncertainty and delayed diagnosis and treatment. Meckel’s diverticulitis in pregnancy is extremely rare with only 27 cases reported in literature, only 11 of which are found to be perforated intraoperatively. We present a rare case of a patient in third trimester of pregnancy with perforated Meckel’s diverticulitis.

## Introduction

Acute abdomen in pregnancy occurs in 1:500 cases [1]. Patients often present with non-specific symptoms such as nausea, vomiting, altered bowel habits and abdominal pain, some of which are normal in pregnancy. Obstetric causes such as pre-term labour, chorioamnionitis, placental abruption or ectopic pregnancy are usually excluded first with assessment in a specialist maternal foetal assessment unit (MFAU). The non-obstetric causes include appendicitis, cholelithiasis, cholecystitis, urolithiasis, pyelonephritis, bowel obstruction, volvulus and intussusception. Anatomical changes during pregnancy such as the growing gravid uterus displacing bowel and other organs as well as physiological changes such as mild to moderate leukocytosis and tachycardia all add to the diagnostic challenge.

Meckel’s diverticulum is the most common congenital malformation of the gastrointestinal tract. It results from the incomplete obliteration of the vitello-intestinal duct leading to the formation of a true diverticulum of the small intestine, involving all three layers [2]. The true prevalence is challenging to define as the majority of patients are asymptomatic. Studies report a prevalence of 1.2–3.0% of the population [2].

Presentations with symptomatic Meckel’s diverticulum during pregnancy are extremely rare. Only 27 case reports of Meckel’s diverticulum can be found in a search of the English literature in the last 30 years [3]. Cases of perforated Meckel’s diverticulum during pregnancy are even rarer, with only 11 cases found [3]. Diagnosis can be difficult and delayed due to the infrequent presentation, the anatomical changes of pregnancy and limited diagnostic tools. Meckel’s diverticulitis can often mimic other causes of abdominal pain such as inflammatory bowel disease and appendicitis.

### Care presentation

A 23-year-old primigravid woman presented at 34 weeks of gestation with sudden onset, severe, right iliac fossa pain that woke her from sleep. It was sharp in nature, did not radiate and was worse with movement. The pain was associated with nausea and vomiting and bowels had not opened for four days. She was admitted to the MFAU where the foetus was confirmed to be healthy. Pre-term labour and ovarian torsion were excluded by the obstetric team.

At the time of the surgical consultant review, the pain was increasingly severe (9/10) despite opioid analgesia. The patient was afebrile with a heart rate of 110 bpm (within normal limits in third trimester) with tenderness and guarding in the right iliac fossa and flank.

### Diagnostic assessment

The results of the initial haematological investigations showed the white cell count was raised at 16.0 (reference value (4.0–11.0) × 10^9^/L) with a neutrophilia and further increased to 20.8 preoperatively. C-reactive protein was normal 2.60 mg/L (reference <30.0 mg/L) and increased to 7.6 mg/L preoperatively.

**Figure 1:**
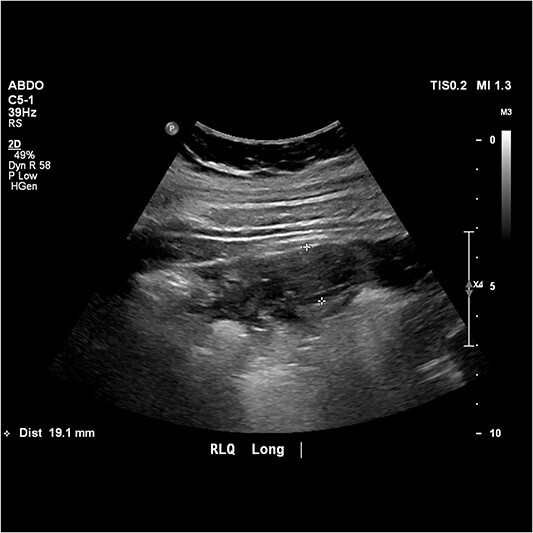
Image from abdominal ultrasound demonstrating hyperaemic, tubular structure measuring 19.1 mm in width with echogenic fat.

Urgent pelvic ultrasound was performed which described a 19 mm blind ending tubular structure with surrounding hyperaemia, echogenic fat and focal tenderness, which was marked by the sonographer in the right upper quadrant and deemed compatible with acute appendicitis (Fig. 1).

Further investigation with CT was considered after discussion with the Obstetric team, given the atypical presentation of appendicitis; however, given the progressive pain and peritonism, and the unequivocal ultrasound findings, the decision was made to proceed to surgery for presumed appendicitis.

### Treatment

**Figure 2:**
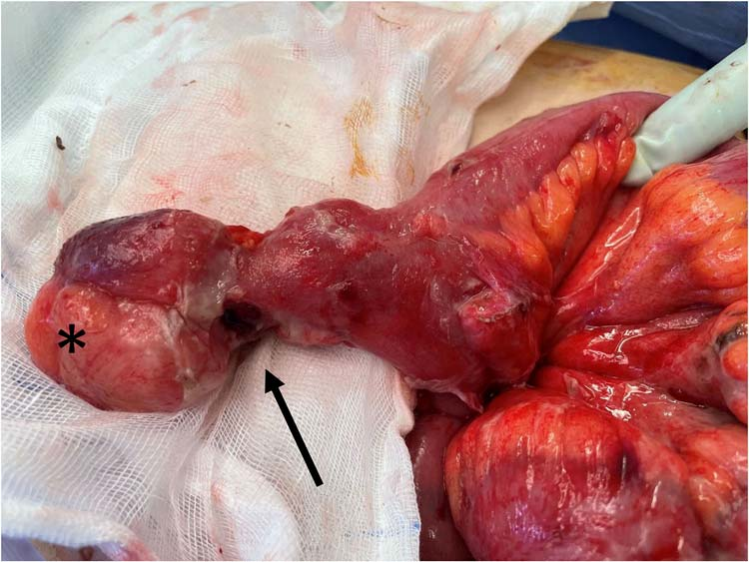
Intraoperative photo showing Meckel’s diverticulum with a mass (*) at the tip and adjacent proximal perforation proximal (black arrow).

The patient was commenced on intravenous antibiotics and proceeded to theatre to have an open exploratory laparotomy via a transverse incision in the right upper quadrant. Intraoperatively, an approximately 7 cm inflamed Meckel’s diverticulum with a thickened mass at the tip and evidence of perforation just proximal to this was evident (Fig. 2). Both the Meckel’s and adjacent small bowel were involved in a local phlegmon with fibrin, adherent to small bowel mesentery. Two enlarged apical lymph nodes were identified and decision was made to complete a small bowel resection including the apical nodes rather than a diverticulectomy. Side to side anastomosis was performed with a linear stapler. The abdominal cavity was washed with warmed normal saline until clear. The right upper quadrant transverse incision was closed in layers with no drain left *in situ*.

### Outcome and follow-up

Postoperative ileus gradually improved, diet was gradually upgraded as tolerated and the patient was discharged home on postoperative Day 6.

The histopathology confirmed Meckel’s diverticulum with diverticulitis. Heterotopic gastric mucosa and associated fibrinopurulent serositis was noted; however, there was no dysplasia or malignancy and the lymph nodes were all reactive.

There were no obstetrics concerns postoperatively. The patient was admitted to MFAU at 40 + 2 weeks gestation, in early labour and had vaginal delivery with smooth postnatal recovery.

## Discussion

### Imaging

It is well known that ultrasound scan (USS) is the preferred imaging modality for investigating the aetiology of acute abdominal pain in pregnancy due to its availability and its lack of ionizing radiation. When USS is inconclusive, magnetic resonance imaging (without intravenous Gadolinium) is recommended as it provides excellent soft tissue views with multiplanar imaging and is safe in any trimester [4]. However, it is not readily available particularly at peripheral hospitals or after hours.

When considering the CT of the abdomen and pelvis in a pregnant patient, a risk-benefit assessment should be performed. Although there is strong evidence that exposure to ionizing radiation in pregnancy increases the risk of teratogenesis and childhood haematological malignancies, the radiation exposure of 20–50 mGy^5^ is less than the recommended maternal dose (50 mGy) below which foetal teratogenesis should not occur [5]. If urgent diagnosis is required and USS has been inconclusive or magnetic resonant imaging (MRI) is not available, then CT can be considered in selective cases.

### Laparoscopy versus laparotomy in pregnancy

Once the decision is made to operate the next consideration is whether to perform laparoscopy versus laparotomy. This is based on the pathology, the size of the gravid uterus, available resources and surgeon preference. We chose an open approach because of the location of the pain in the right upper quadrant, the gravid uterus measuring more than 34 weeks and suspected perforation with clinical peritonitis. In this particular case, we found perforated Meckel’s diverticulum and we were able to resect and washout via the open approach. However, laparoscopy is considered to be safe during any trimester without any increased risk to mother or foetus and the benefits include less postoperative pain, decreased risk of foetal respiratory depression secondary to opioid analgesia, less wound complications, less postoperative ileus and decreased length of stay [4, 6].

## Conclusion

Meckel’s diverticulitis is an important consideration in pregnant patients presenting with acute abdomen and can often be misdiagnosed as appendicitis. Ultrasound should be used for initial diagnostic imaging; however, MRI without gadolinium contrast and CT can be considered if available and if there is diagnostic uncertainty. The treatment of Meckel’s diverticulitis is always surgical intervention, which can be performed both open or laparoscopically, depending on the fundal height and surgeon’s expert opinion.

## Data Availability

There is no data as this is a case report.
